# Berry Phenolics of Grapevine under Challenging Environments

**DOI:** 10.3390/ijms140918711

**Published:** 2013-09-11

**Authors:** António Teixeira, José Eiras-Dias, Simone D. Castellarin, Hernâni Gerós

**Affiliations:** 1 Center for the Research and Technology of Agro-Environmental and Biological Sciences (CITAB), Quinta de Prados, 5001-801 Vila Real, Portugal; E-Mail: antonio.teixeira@bio.uminho.pt; 2 Department of Biology, School of Sciences, University of Minho, Campus de Gualtar, 4710-057 Braga, Portugal; 3 National Institute of Agrarian and Veterinary Research (INIAV), Quinta da Almoinha, 2565-191 Dois Portos, Portugal; E-Mail: eiras.dias@inrb.pt; 4 Department of Agrarian and Environmental Sciences, University of Udine, via delle Scienze 208, 33100 Udine, Italy; E-Mail: simone.castellarin@uniud.it

**Keywords:** agricultural practices, environmental challenges, genotype and environment interactions, grape berry phenolics, varietal diversity, *Vitis vinifera*

## Abstract

Plant phenolics have been for many years a theme of major scientific and applied interest. Grape berry phenolics contribute to organoleptic properties, color and protection against environmental challenges. Climate change has already caused significant warming in most grape-growing areas of the world, and the climatic conditions determine, to a large degree, the grape varieties that can be cultivated as well as wine quality. In particular, heat, drought and light/UV intensity severely affect phenolic metabolism and, thus, grape composition and development. In the variety Chardonnay, water stress increases the content of flavonols and decreases the expression of genes involved in biosynthesis of stilbene precursors. Also, polyphenolic profile is greatly dependent on genotype and environmental interactions. This review deals with the diversity and biosynthesis of phenolic compounds in the grape berry, from a general overview to a more detailed level, where the influence of environmental challenges on key phenolic metabolism pathways is approached. The full understanding of how and when specific phenolic compounds accumulate in the berry, and how the varietal grape berry metabolism responds to the environment is of utmost importance to adjust agricultural practices and thus, modify wine profile.

## 1. Introduction

**Figure 1:**
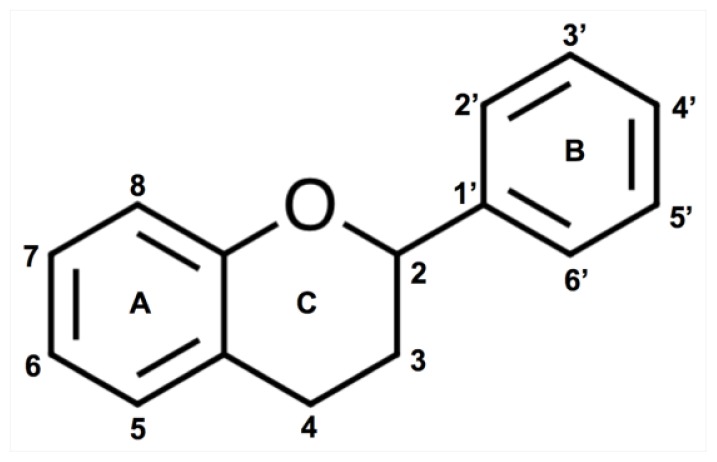
Flavonoid ring structure and numbering.

Phenolic compounds can be defined as molecules naturally derived from plants or microbes, consisting of a phenyl ring backbone with a hydroxyl group or other substitutes. Phenolic compounds of the grape are divided between nonflavonoid (with a simple C6 backbone; hydroxybenzoic acids, hydroxycinnamic acids, volatile phenols and stilbenes) and flavonoid compounds (flavones, flavonols, flavanones, flavan-3-ols and anthocyanins). Nonflavonoid phenolics are found in grapes and wine, but with the exception of hydroxycinnamic acids, they are present in low concentrations [1,2]. Flavonoids make up a significant portion of the phenolic material in grapes and include several classes [2]. They are C6–C3–C6 polyphenolic compounds, in which two hydroxylated benzene rings, A and B, are joined by a three-carbon chain that is part of a heterocyclic C ring (Figure 1). According to the oxidation state of the C ring, these compounds are divided into structural classes that include flavonols, flavan-3-ols (that include simple flavan-3-ols and their polymeric forms proanthocyanidins), and anthocyanins [3].

Grape phenolics contribute to color, flavor, texture and astringency of wine and to its antioxidant properties. The biosynthesis of soluble phenolics begins with the aromatic amino acid phenylalanine, a product of the shikimate pathway. The early precursors of the shikimate pathway are erythrose-4-phosphate and phosphoenol pyruvate. This pathway is responsible for producing phenylalanine and the other amino acids tyrosine and tryptopahne [2,3]. Although the biosynthesis of many secondary compounds has been elucidated in detail, reports on the identification of transporters of secondary compounds have been published only recently [4,5] and a clear and precise understanding of flavonoid transport in plants is far from being elucidated.

**Figure 2:**
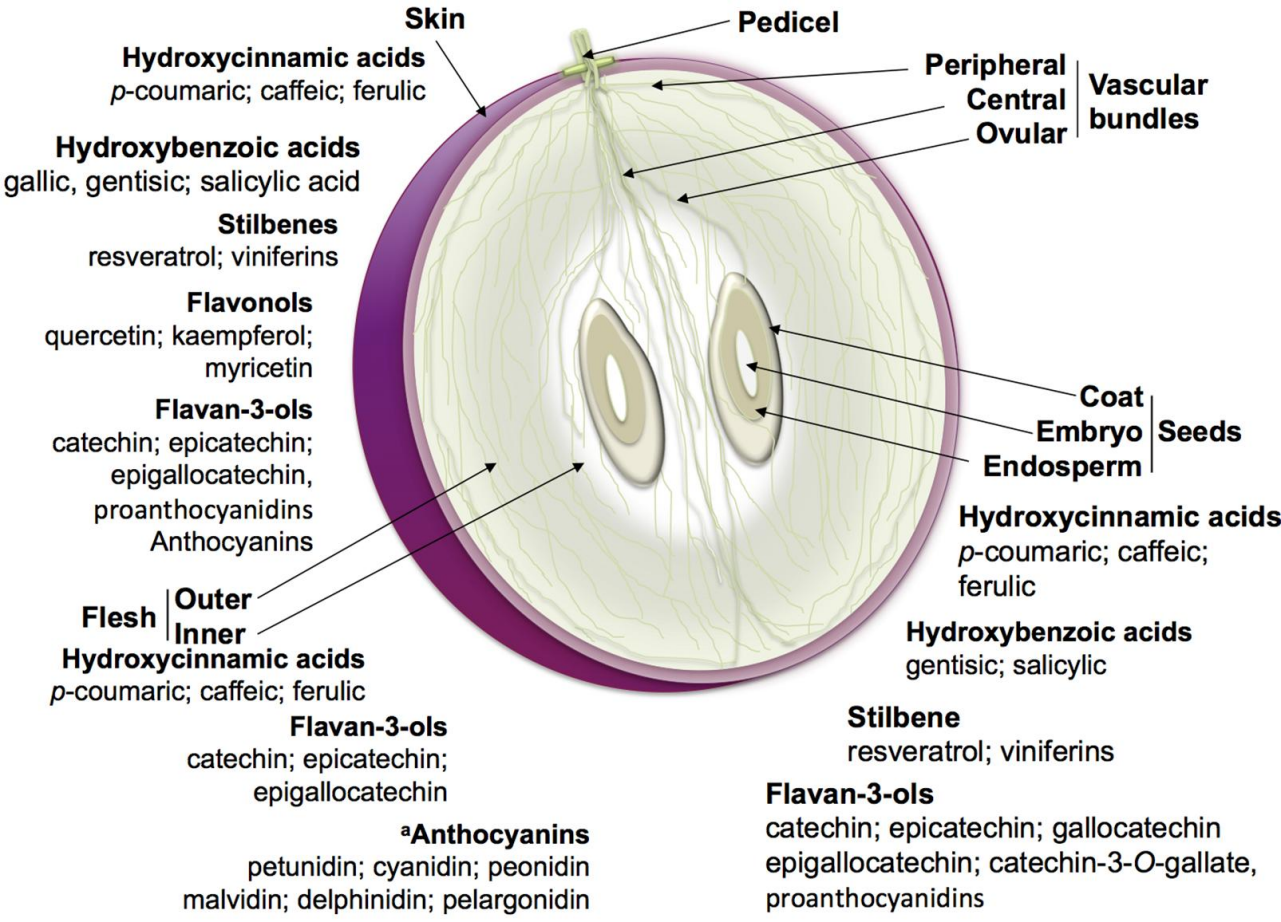
Schematic structure of a ripe grape berry and pattern phenolics biosynthesis distribution between several organs and tissues (indicated by arrows). ^a^ Anthocyanins are synthetized also in the inner flesh of the teinturier varieties [2,6–12].

Two distinguishable tissues compose the grape skin, representing the hydrophobic barrier of the pericarp. The outermost—the epidermis—is strongly cutinized, while the inner thick-walled layers of hypodermis (assumed to consist of several layers, depending on the variety), contain most of the skin flavonoids. In this fraction, the major class of flavonoids is represented by anthocyanins, proanthocyanidins and, to a minor extent, simple flavan-3-ols and flavonols [4]. A schematic structure of a ripe grape berry with the distribution pattern of secondary metabolites between tissues is shown in Figure 2.

While there is debate about the anthropogenic influence on climate, there are clearly recorded periods of extreme temperature events that may have implications for grape cultivation and wine quality [13–16]. Climate change imposes rapid drifts in weather patterns that determine the suitability of growing regions for specific types of wine [17]. Climate changes in the future might extend the north and south latitude boundaries of areas where good wines are produced [18]. However, some areas that nowadays are producing high quality grapes may be affected by heat and water stress [17]. The climate changes are particularly important for grapevine cultivation, in which heat, drought and light intensity are just some environmental stress factors that dramatically affect phenolic metabolism as well as grape development and chemical composition. In this regard, cultural practices, such as canopy management and irrigation may be optimized to adjust berry and wine quality.

Nowadays, the genetic diversity conservation of grapevine is a big concern. The genus *Vitis* contains more than 70 species growing widely in distinct geographical areas [19]. The most renowned species is *Vitis vinifera* that was domesticated in Asia Minor or Armenia 5000 years ago, from where it spread to other countries. The high morphological and genetic diversity of vinifera has an estimated number of more than 10,000 cultivars. While many factors, such as viticulture practices, environmental conditions, and post-harvest processing activities, can all affect the content of total polyphenols or individual polyphenolic compounds in grapes and grape products, varietal or genetic difference is one of the most important factors [20]. This review deals with the diversity and biosynthesis of phenolic compounds in the grape berry, from a general approach to a more detailed level, such as the influence of the environmental factors, including drought and heat, and the genotype dependence on the production of grape phenolics. The comprehension of how and when specific phenolic compounds accumulate in the berry, and how the grape berry metabolism responds to the environment is of utmost importance to adjust agricultural practices and thus, modify wine profile.

## 2. Metabolism and Compartmentation of Phenolics in the Grape Berry

### 2.1. Nonflavonoid Phenolics

The hydroxycinnamates are the third most abundant class of soluble phenolics in grape berries, after proanthocyanidins and anthocyanins. Phenolic hydroxycinnamates are commonly accumulated in berry skin and the flesh of white and red *vinifera* and *non-vinifera* varieties [21]. Thus, while they are also found in red wines, they are usually the most abundant class of phenolics in free-run juice and white wines where they contribute to colour browning under oxidation with non-phenolic molecules [1,2,6,22]. In terms of concentration, *p*-coumaric, caffeic and ferulic acids are also predominant phenolics in grape. These three hydroxycinnamic acids are present primarily as *trans* isomers, although traces of *cis* isomers have been detected. They differ by the type and number of substituents on the aromatic ring. When these hydroxycinnamic acids are esterified with tartaric acid, they are named coutaric acid (*trans*-*p*-coumaroyl-tartaric acid), caftaric acid (*trans*-caffeoyl-tartaric acid), and fertaric acid (*trans*-feruloyl-tartaric acid) [3].

The synthesis of hydroxycinnamates occurs mainly before *veraison* (Table 1). During ripening, their concentration decreases with the increasing fruit size and dilution of solutes, though its content per berry remains almost constant. Although its accumulation occurs predominantly in the flesh they are present in all berry tissues [4,13] (Figure 2 and Table 1). In hypodermal, mesocarp and placental cells of the pulp, hydroxycinnamates may be conjugated with anthocyanins [2,3,13].

The levels of hydroxybenoic acids and their derivatives are commonly low in wine, compared to the levels of hydroxycinnamic acids. The most common hydroxybenzoic acids in grape berry include gentisic acid, salicylic acid, gallic acid, and *p*-hydroxybenzoic acid, which are mainly found in their free form [23–25]. Gentisic acid is accumulated at very low levels, as is salicylic acid which is involved in signaling in plants, particularly in the induction of defense and stress responses [3,25]. The most represented is gallic acid, which is found free as well as acyl substituent of flavan-3-ols. Other benzoic acids such as protocatechuic, vanillic and syringic acids are found in Riesling wine from Germany [26]. In the seeds, gallic acid can esterify the carbon in position 3 of flavan-3-ols [6].

A nonflavonoid compound class that, although present in trace quantities in wine, has been drawing attention is stilbenes [2]. These compounds occur naturally in a few edible plants, and several species of the genus *Vitis* are proficient at stilbenes synthesis, mainly in the skin at the mature stage (Table 1 and Figure 2). Stilbene content of the berry changes across varieties [7]. Their synthesis also increases upon pathogen infection and in response to abiotic stress [8]. Some stilbenes, particularly resveratrol, have been drawing attention for their benefits to human health. Stilbenes can undergo glycosylations or methylations. Glycosylated resveratrol originate piceids, *trans*- and *cis*-resveratrol-3-*O*-β-d-glucopyranosidade as well as astringin, which is a 3′-OH-*trans*-piceid. Modifications by addition of two methyl groups to the resveratrol originate pterostilbene (3,5-dimethoxy-4′-hydroxystilbene) with enhanced antifungal activity compared to the non-methylated form [35].

*Trans*-resveratrol (3,5,4′-trihydroxytilbene) is the stilbene with the simplest molecular structure, which is used as precursor for other compounds through various modifications of the stilbene unit. *Cis*-resveratrol is a *trans*-resveratrol isomer although less stable [35]. Oligomerisation of stilbenes can be derived in dimers, trimers and tetramers from oxidative coupling of resveratrol and derivatives by 4-hydroxystilbenes peroxidases. Viniferins are a major group of resveratrol oligomers produced by oxidation of basic stilbenes. The most important viniferins are α- β- γ- δ- ɛ-viniferins, composed essentially by cyclic oligomers of resveratrol [3].

### 2.2. Flavonoids

From an anatomical point of view, grape flavonoids are localized mainly in both the peripheral layers of berry pericarp (skin) and in some layers of the seed coat. Most of the skin flavonoids are abundant in the inner thick-walled layers of hypodermis. In this fraction, the major class of flavonoids is represented by anthocyanins, proanthocyanidins (also known as tannins) and, to a minor extent, simple flavan-3-ols and flavonols [4,6] (Figure 2 and Table 1).

Flavonols are a class of flavonoids with a 3-hydroxyflavone backbone. They differ by the number and type of substituents on the B ring (see Introduction), and occur conventionally as glucosides, galactosides, rhamnosides and glucuronides with the sugar bond attached to the 3 position of the flavonoid skeleton. The grape berry synthetizes kaempferol, quercetin, myrcetin and the methylated forms isoharmnetin, laricitrin and syringetin [36]. Flavonols constitute the third component of flavonoids in the skin fraction (Table 1). Quercetin is known to behave as UV-protectant and to play a role in co-pigmentation with anthocyanins [4]. As reported below, flavonol concentration varies extensively among varieties, ranging from 0.018 mg to 0.176 mg per g of berry FW, but its content in the berry can be strongly affected by environmental factors, particularly sunlight exposure (among the others, see [20,28,37]. Flavonol synthesis occurs primarily during early stages of fruit development and ends at around *veraison* [28] (Table 1).

Flavan-3-ols are the most abundant class of phenolics in the grape berry [38]. They have a monomeric (catechins) or polymeric structure known as proantocyanidins or condensed tannins. Catechins and proantocyanidins are located essentially in the seeds, then in the skins and very little in the pulp [39]. Catechins are responsible for bitterness in wine and may also be partially associated with astringency [1,2,6]. The five flavan-3-ols in grapes are (+)catechin and its isomer (−)epicatechin, (+)gallocatechin, (−)epigallocatechin and catechin-3-*O*-gallate. Catechins are characterized by the presence of a hydroxyl group at the 3 position of the C ring [2,3,22,40].

Proantocyanidins are a diverse group of compounds composed by flavan-3-ols polymer subunits that are linked via 4–6 and 4–8 interflavan bonds. These phenolic compounds are the most abundant class of soluble polyphenols in grape berries. Proanthocyanidins vary in size, ranging from dimers to polymers with more than 40 units [2,3,28,41].

Flavan-3-ols are detectable in highest concentration in seeds (Figure 2 and Table 1). Proanthocyanidins are predominantly found in the hypodermal cell layers of the berry skin and in the soft parenchyma of the seed coat inside the vacuole or bound to cell wall polysaccharides [1–3,6]. Grape proanthocyanidins have a larger average size in the skin than in the seeds. These proanthocyanidin compounds are responsible for the grape skin organoleptic properties such as astringency and bitterness in grape skin or wine [2,4].

Anthocyanins are responsible for red, purple and blue pigmentation of the grape berries and, consequently, the red wine. The structures of the common anthocyanins in *V. vinifera* grapes and wine were determined in 1959 [2,42]. The core of the anthocyanidin, the flavylium, has the typical C6–C3–C6 skeleton. Intrinsically, anthocyanins are glycosides and acylglycosides of anthocyanidins, and the difference of the aglycones and flavyliums (2-phenylbenzopyrilium) occurs at the 3′ and 5′ positions of the B ring, due to hydroxyl or methoxyl substitutions [43]. Anthocyanins can also be esterified by acids, such as acetic, coumaric or caffeic, linked to the 6′ position of the glucose bonded to the 3′ position of the C ring [2,6]. There are 17 naturally occurring aglycones, but only six are reported in grapevine: malvidin, cyanidin, peonidin, delphinidin and petunidin. Traces of pelargonidin are found in Pinot Noir and Cabernet Sauvignon [44], but the malvidin-3-*O*-glucoside was found to be the major anthocyanin present along with its acylated forms [2]. *V. vinifera* contains only 3-*O*-monoglycosides due to two mutations in the 5-*O*-glucosyltransferase gene which implicated the loss of the dominant allele involved in the production of diglucosidic anthocyanins [43,45,46]. The anthocyanins commonly found in *V. vinifera* grape include delphinidin, cyanidin, petunidin, peonidin and malvidin 3-glucosides, 3-(6-acetyl)-glucosides and 3-(6-*p*-coumaroyl)-glucosides, peonidin and malvidin 3-(6-caffeoyl)-glucosides, being that malvidin-3-*O*-glucoside is generally the major anthocyanin present along with its acylated forms (Figure 2).

Differently from proanthocyanidin, accumulation of anthocyanin pigments in red grape varieties starts from *veraison* and reaches its maximum in the latest phases of fruit maturation when the synthesis stops (Table 1). Anthocyanins are synthesized in the cytosol of the epidermal cells, are co-localized with proanthocyanidins in the skin hypodermal layers and then stored in the vacuole [4,9] (Figure 2 and Table 1). In a few teinturier varieties, accumulation in the berry skin is paralleled by accumulation in flesh [3,4,47]. In the red flesh variety Alicante Bouschet, colour development began in the flesh at the stylar end of the fruit and progressed toward the pedicel end flesh and into the skin [10].

### 2.3. Biosynthesis Pathways of Phenolic Compounds in Wine Grape

**Figure 3:**
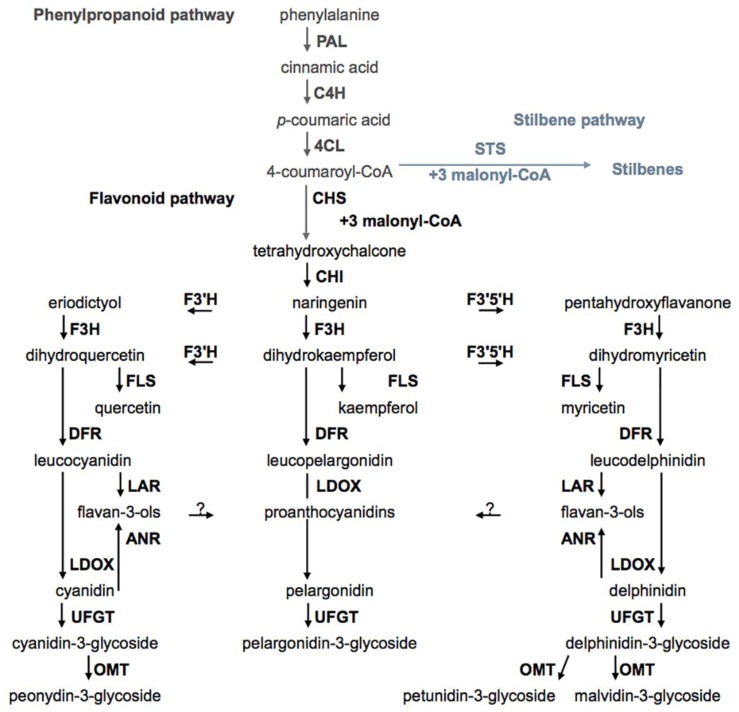
Biosynthetic pathways of grape berry secondary compounds. Phenylalanine ammonia lyase (PAL), cinnamate-4-hydroxylase (C4H), 4-coumaroyl:CoA-ligase (4CL), stilbene synthase (STS), chalcone synthase (CHS), chalcone isomerase (CHI), flavonoid 3′-hydroxylase (F3′H), flavonoid 3′,5′-hydroxylase (F3′5′H), flavanone-3-hydroxylase (F3H), flavonol synthase (FLS), dihydroflavonol reductase (DFR), leucoanthocyanidin reductase (LAR), anthocyanidin reductase (ANR), leucoanthocyanidin dioxygenase (LDOX), dihydroflavonol 4-reductase (DFR), flavonoid glucosyltransferase (UFGT), *O*-methyltransferase (OMT) (adapted from [3,43]).

The biosynthetic pathways of different phenolics have been recently thoroughly reviewed by Castellarin *et al.* [3] and He *et al.* [43] and are schematically presented in Figure 3.

Hydroxycinnamic acids are generated by modifications to intermediates of the phenylpropanoid pathway. First reaction synthesis of simple phenolics in grape involves the deamination of phenylalanine by the enzyme phenylalanine ammonia lyase (PAL), in which the product is cinnamic acid [48]. The enzyme cinnamate-4-hydroxylase (C4H) converts cinnamic acid to *p*-coumaric by hydroxylation. *p-*coumaric is esterified by the enzyme CoA-ligase (4CL) producing 4-coumaroyl-CoA. In these modifications, 3-hydroxylation of *p*-coumaric originate caffeic acid, which can be converted into ferulic acid by 3-methylation. This product is substrate of two enzymes, chalcone synthase (CHS) and stilbene synthase (STS).

The first step of the stilbene pathway is controlled by STS. The competition of STS and CHS for the same substrate, 4-coumaroyl-CoA, controls the entry point into the stilbene pathway and flavonoid pathway. In an analogous way of CHS, STS carry out three reactions of condensation that produce resveratrol. Although, in the STS reaction, the terminal carboxyl group is removed prior to closure of the A ring, causing a different ring-folding in resveratrol compared to the CHS product tetrahydroxychalcone.

All flavonoids stem from tetrahydroxychalcone. The flavonoid pathway leads to the synthesis of different classes of metabolites such as flavonols, flavan-3-ols, proanthocyanidins, and anthocyanins (Figure 3).

Some mechanisms have been proposed concerning flavonoid transport in plants. Flavonoid uptake across the tonoplast may be mediated by a primary active transport, driven by ABC proteins. Very recently it was shown that the ABC protein ABCC1 that localizes to the tonoplast is involved in the transport of glucosylated anthocyanidins, which depends on the presence of GSH but not on the formation of an anthocyanin-GSH conjugate [49]. ABCC1 is expressed in the exocarp throughout berry development and ripening, with a significant increase at *veraison*. A genetic screen aimed to study flavonoid biosynthesis provided the first evidence for the involvement of MATE proteins in the transport of flavonoids across the tonoplast. MATE transporters are highly upregulated during maturation, the time when grape berries start to accumulate anthocyanins. It has also been suggested that flavonoid moieties, depending also on their different substituting groups (acyl, glycosyl and/or methoxyl), are driven to their accumulation sites by a complex vesicle trafficking system involving the Golgi apparatus [4]. The two grape berry MATEs, anthoMATE1 (AM1) and AM3, specifically transport acylated anthocyanins [50,51]. Subcellular localization assays revealed that anthoMATE transporters were closely related with these small vesicles, whereas GST was localized in the cytosol around the nucleus, suggesting an association with the endoplasmic reticulum [52]. While the biosynthesis and regulation mechanisms of anthocyanin synthesis have been extensively studied, the knowledge on the mechanisms of their sequestration in the vacuole and to what extent their color is affected by vacuole storage is still limited.

## 3. Impact of Environment and Agricultural Practices in Grape Berry Phenolics

**Figure 4:**
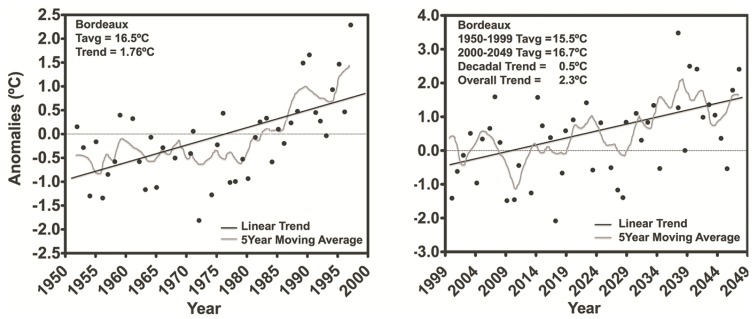
HadCM3 modeled growing season average temperature anomalies for the Bordeaux region. The anomalies are referenced to the 1950–1999 base period from the HadCM3 model. Trend values are given as an average decadal change and the total change over the 2000–2049 50-year period. Note: this figure is adapted with permission from [56]. Copyright Springer, 2005.

Several regional climate models have been proposed in order to forecast the overall effects of individual or combined climate change-related variables [53]. Some models take into account air temperature and other variables, including precipitation, humidity, radiation, and historical viticultural records [54]. Spatial modeling research has indicated potential geographical shifts and/or expansion of viticultural regions with parts of southern Europe becoming too hot to produce high-quality wines and northern regions becoming viable [17,18,55]. For the Northern hemisphere, Jones *et al.* [56] predicted that temperatures at regions producing high-quality wine between 2000 and 2049 are going to warmby 0.42 °C per decade and 2.04 °C overall. In the Bordeaux region, the predicted increase temperature overall trend would be 2.3 °C in the same period (Figure 4).

For vineyards, the increase in the number of days with high temperatures is particularly relevant. Grape production and quality are sensitive to heat waves, especially at certain growth stages, such as flowering and ripening. At high temperatures, replacement of starch by lipids in leaf chloroplasts has been reported for grapevines [57]. Prolonged periods with temperatures above 30 °C cause a reduction in photosynthesis, with consequent berry size and weight reduction [58]. High temperature conditions may have implications in premature *veraison*, berry abscission and reducing flavour development.

Metabolic processes and sugar accumulation, beyond other parameters related to colour and aroma, may also be affected or completely stopped by high temperatures [11,59,60].

Studies carried out in European countries have highlighted harvest date advances associated with temperature increases. In southern France, the harvest dates advanced by between 18 and 21 days from 1940 to 2000 [61] and in Alsace (eastern France) the harvest was two weeks earlier in 2002 than in 1972, a period during which temperature increased by 1.8 °C [62].

In the viticultural French region of Languedoc, the climacteric evolution over the period 1950–2006 obeyed to two distinct climate periods, according to Laget *et al.* [63]. Observing the evolution of mean annual and seasonal temperatures, total solar radiation, night freshness index, the distribution and efficiency of rainfall and potential evapotranspiration (pET), it was reported an increase in mean annual temperatures of +1.3 °C between 1980 and 2006 and an increase in the mean pET of 900 mm/year since 1999. It was also concluded that the harvest dates advanced by up to three weeks and sugar concentrations at harvest increased by up to 1.5% potential alcohol. In the Bordeaux region, from 1952 to 1997 changes in the dates of all the phenological events and in the length of the growing season were reported for Cabernet Sauvignon and Merlot [64]. Similar results were found in the southern hemisphere. In Australia, the date of designated maturity of Chardonnay, Cabernet Sauvignon and Shiraz advanced at rates of between 0.5 and 3.1 days per year between 1993 and 2006 [65]. A trend towards earlier maturity of several varieties was observed in 12 different Australian winegrape growing regions form 1993–2009 [66]. For most of the cases, the rate of change in the date of designated maturity was correlated with the rate of change in temperature.

### 3.1. Temperature and Radiation

Of environmental factors including all external stimuli, the most influential of which for phenolic synthesis are light/radiation and temperature, as well as water and nutritional status. Phenolic synthesis and accumulation in grape berry is also determined by genetic factors and the interaction between genotype and environment [3,53]. The role of phenolics as photo-protectants explains their dependency on sun exposure [53]. In warmer climates, high light exposure can increase the concentration of phenolics and anthocyanins because of the higher activity of PAL [67]. Sun exposure is generally considered to be of primary importance for high quality wine production. However, it is not clear whether the effect on fruit composition is due to visible light or ultraviolet light or both [68,69].

It has been shown that UV-B provoke several morphological, physiological and biochemical changes in higher plants, depending on the intensity, total dosage, plant species and the balance between UV-B and photosynthetically active radiation (PAR, 400–700 nm) [69,70]. On the other hand, UV-A and visible light may induce both protective and repair mechanisms, thus decreasing the negative impact of UV-B light [71]. However, relatively high levels of solar UV-B were reported to enhance the accumulation of UV-absorbing compounds, including flavonoids and related phenolics [72]. UV-B is also known to upregulate genes encoding PAL and CHS [70]. Phenolics transform short-wave, high-energy and highly destructive radiation into longer wavelength light, less destructive to the cellular leaf structures, including the photosynthetic apparatus [69]. Very few studies have attempted to separate the effects of visible light from those of UV light [59,73]. As discussed by Keller [74], this is surprising given that phenolic compounds are absorbed predominantly in the UV range of the spectrum and form an important part of fruit quality in grapes.

Stilbene synthesis is enhanced in response to several abiotic factors. These factors include UV-radiation, wounding, ozone, anoxia and metal ions. Exposure to UV light induces the accumulation of stilbenes in grape berry through the induction of STS expression [75]. In berries, this is dependent on the development stage, since unripe berries respond to UV irradiation to a greater extent. A study on grape plantlets proved the existence of a positive correlation between resveratrol synthesis in leaves (induced by UV) and field resistance [76].

Flavonols are thought to protect plant tissue to UV radiation whereas anthocyanins are thought to provide some protection to UV radiation and high extreme temperatures [6]. Synthesis of flavonols is a light-dependent process. Sealing grape bunches in light-excluding boxes from before flowering until harvest completely inhibits flavonol synthesis. If shading is applied later in fruit development, flavonol content is reduced and no further accumulation is detected after the initiation of light deprivation [3,6,37,77,78]. In Pinot Noir, Shiraz, and Merlot varieties, the amount of these compounds has been shown to be highly dependent on light exposure of the tissues in which they accumulate [78]. Light modulates the expression of *flavonol synthase* (*VvFLS*), a key flavonol structural gene*,* and of *VvMYBF1*, a transcriptional regulator of flavonoid synthesis [79–81]. In Cabernet Sauvignon and Chardonnay, flavonols are the only phenolic components in both grape leaves and berries that are consistently and severely increased by UV radiation [68]. It was suggested that flavonols, but not anthocyanins or hydroxycinnamic acids, are important for UV protection in grapevine tissues. Similar results were recently confirmed by Koyama *et al.* [81] who showed that UV light specifically induced flavonols while not affecting other flavonoid components. However, the relatively high concentrations of flavonols found even in the absence of UV radiation suggest that flavonols may also have a protective function against excess visible radiation [68]. In the vineyard, any cultural practices that favor the exposure of grape brunches to sunlight boost flavonol accumulation. This occurs equally in white and red grapes.

Flavan-3-ols and proanthocyanidins are the most stable phenolics under diverse growing conditions. This is also true for accumulation of these compounds in seeds. However, some studies have shown a positive association between temperature and the number of seeds and total proanthocyanidin levels per berry at harvest [82,83]. Shading treatments increased the amount of seed proanthocyanidins and affected their composition in Pinot Noir [84], while had no effects in Shiraz [78], reiterating the importance to discriminate between irradiation and temperature effects [53].

Skin flavan-3-ols and proanthocyanidins are more sensitive than seed ones to environmental cues; sunlight has been shown to affect their relative content [78,81,84], as well as their mean degree of polymerization [81,84]. Sunlight exposure consistently increased the relative abundance of the tri-hydroxylated gallocatechins at the expense of the di-hydroxylated catechins and increased the mean degree of polimeryzation.

When the effect of cluster temperature on proanthocyanidins biosynthesis was studied it was shown that there is no consistent relationship between temperature and total proanthocyanidins accumulation across three seasons [16]. In this field, experiment grape bunches were cooled during the day and heated at night (±8 °C). However, composition of proanthocyanidins was affected in the experiment because decreasing thermal time in degree-days favored a shift towards tri-hydroxylated forms.

Although anthocyanins and proanthocyanidins share several steps in the biosynthetic pathway, there are many differences in their regulation and reactivity. In fact, in contrast with proanthocyanidins, several authors reported that light, temperature, and their interactive effects, highly influence anthocyanin accumulation in berry skins [85,86]. Exposure to sunlight is associated with an increase in anthocyanin accumulation, until the point when excessive heat causes berry temperature to become detrimental [3,77,87]. In growth chambers, optimal conditions for anthocyanin accumulation occurred when grapes were exposed to cool nights (15 °C) and mild, temperate days (25 °C) during ripening [88]. Higher temperatures (30–35 °C) promote the degradation of the existing anthocyanins [89]. In the Merlot variety, attenuation of the diurnal temperature fluctuations led to increased ripening rates and higher anthocyanin concentrations at harvest [90]. Moreover, absolute anthocyanin levels and chemical composition changes have also been related with warmer seasons, as indicated by the increased formation of malvidin, petunidin, and delphinidin coumaroyl derivatives [85]. In another study [87], the association of high temperatures with the increase of delphinidin, petunidin and peonidin-based anthocyanins in sun-exposed Merlot berries were observed, while malvidin derivatives remained unaffected. The complexity of combined solar radiation and temperature effects on flavonoid composition further expands the understanding of the effect of such environmental factors on anthocyanin biosynthesis [53].

### 3.2. Agricultural Practices and the Levels of Synthesized Metabolites

In a vineyard, the environment varied due to the natural soil heterogeneity and the uneven light distribution. Physical characteristics of the vineyard can also affect flavonoid accumulation. These include altitude of the cultivation site, heat stress, defoliation, mineral supply or soil type, all of which have shown some influence. Nitrogen, potassium and phosphate are the nutrients commonly applied as fertilizers, although only nitrogen and potassium have thus far attracted viticultural research. Both low and excessively high levels of nitrogen have been shown to decrease color in grape berries, while high potassium has been reported to decrease color in grapes [85,91,92]. Despite the age of the soil, which largely determine the micronutrient pool, structure and texture, and significantly affects plant growth [93–95], the major consequence of soil type is the capacity of the soil to hold water while remaining sufficiently well-drained to avoid waterlogging [85,96,97].

Despite the relevance of these parameters, vineyard microclimate has a fundamental influence in the metabolite biosynthesis. The importance of the effect of canopy microclimate on chemical composition of berry was initially raised by Shaulis and co-workers [98] in their investigations with Concord grapevines. The amount and the distribution of light intercepted by the vines are determined by the architecture of the vineyard, mainly row orientation, height, width, porosity of the canopy, and distance between rows [99]. The term “microclimate” was adopted by Smart [100] to define the environmental conditions within the immediate vicinity of the leaves and fruit [101].

Cultural practice effects on berry have long been studied; among them, leaf removal and cluster thinning, which modify leaf area/yield ratio and fruit-zone microclimate, could potentially improve grape quality [86,96,102,103]. The amount of intercepted light affects the whole plant photosynthetic capacity, water balance, and source to sink balance [99,104]. The source to sink balance is an important parameter that controls berry sugar, organic acids, and secondary metabolites content with qualitative enological potential [105]. In general, berries grown under open canopy conditions, compared to berries grown under shaded canopy conditions, have higher juice sugar concentration (measured as total soluble solids), improved acid balance (lower juice pH and higher titratable acidity). However, while some exposure to light may be appropriate, high temperatures resulting from full exposure of berries are likely to inhibit anthocyanin metabolism [101].

Vine vigor has been reported to impact upon the proanthocyanidins content and chemical composition of grape skins in Pinot noir. In the berry skin, proanthocyanidins were higher in low-vigor vines, with an increase in the proportion of epi-gallocatechin subunits, as much in polymers as on average size, observed with decreasing vine vigor [85,106]. It seems that severe canopy shade down regulate gene expression in the anthocyanin biosynthesis pathway, [107,108] while photon fluxes of 100 mmol/m^2^/s on the berries temperature becomes the overriding variable in anthocyanin synthesis [74,77,85,87].

Among environmental and viticultural parameters investigated in the past decades for various grape varieties, it is known that the water status is a potential modulator of secondary metabolism during the berry development [109–112]. Many scientific articles have extensively reported the effects of water deficit on the accumulation of various grape secondary metabolites (Table 2). Grapevine irrigation can alleviate water-stress-related reductions in plant growth and development, demonstrating the importance of cultural practice at vineyard to guarantee wine quality or even plant survival in regions affected by seasonal drought [113]. Several reports demonstrated that large fluxes of water are not essential for the optimal plant performance for agricultural purposes and that moderate water deficits might be used successfully in grapevine production through control of sink-source relationships, thereby maintaining or ameliorating fruit quality [113]. Plant water status affects berry composition, but the effects might be contrasting according to the level and the moment in time when water is applied or deficit is imposed. Furthermore, grape response to moderate irrigation might also be cultivar-dependent as *V. Vinifera* varieties have been shown to respond differently to water stress [114]. Overall, regulation of grapevine water deficit is a powerful tool to manage the amount of secondary metabolite compounds and improve wine quality [115].

The impact of water on stilbene biosynthesis in grapes has been evaluated. The water deficit increases the specific steady state transcript abundance of a STS gene and phenylpropanoid metabolism in general. The increase of STS mRNA abundance suggests an increase in resveratrol accumulation [116]. However, conflicting results have been reported on the effects of water deficit on resveratrol synthesis. Research conducted by Vezzuli *et al.* [117] observed little effect of drought on resveratrol concentrations in grape berry skin. In another study on Cabernet Sauvignon and Chardonnay varieties, harvested at six and eight weeks after *veraison*, respectively, Deluc *et al.* [118] demonstrated that water deficit increased the accumulation of *trans*-piceid (the glycosylated form of resveratrol) by five-fold in Cabernet Sauvignon berries but not in Chardonnay. However, the abundance of two stilbene-derived compounds—*trans*-piceid and *trans*-resveratrol—was not significantly different between the two cultivars when well-watered. Similarly, water deficit significantly increased the transcript abundance of genes involved in the biosynthesis of stilbene precursors in Cabernet Sauvignon. In contrast, the transcript abundance of the same genes declined in Chardonnay in response to water deficit.

The increased concentration of flavonols, skin-derived proanthocyanidins and anthocyanins has also been observed in wines from grapes grown under the decreased vine water status [85,115].

Recently, it was shown that the concentrations of flavonol increase under drought stress in a white grapevine Chardonnay, but not in a red grapevine Cabernet Sauvignon [119]. Few studies have reported that water deficit may modify the skin proanthocyanidins [120–123], but this topic still awaits further clarification. In Shiraz, the application of water stress before and after *veraison* differently affects the grape berry polyphenol biosynthesis [124]. The authors showed that pre-*veraison* water deficit had no effect on total proanthocyanidin accumulation, whereas pre- and post-*veraison* deficits specifically affected the flux of anthocyanin biosynthesis in stressed grape berries sampled with equivalent sugar content. However, both water deficits differently affected the anthocyanin composition. Pre-*veraison* water deficit increased anthocyanin accumulation except for malvidin and *p*-coumaroylated derivatives, whereas post-*veraison* water deficit enhanced the overall anthocyanin biosynthesis, particularly malvidin and *p*-coumaroylated derivatives. In Merlot variety under water stress, an increase of anthocyanin content between 37% and 57% for two consecutive years was reported by Castellarin *et al.* [125].

Imposing water deficits from the onset of ripening until maturity in the Merlot variety reduced the berry weight and increased the concentration of anthocyanins and skin tannins [126], and the application of water deficits also modulated chemical composition changes during berry ripening [125,127].

When Aragonez (Syn. Tempranillo) grapevines were subjected to three irrigation regimes (conventional sustained deficit irrigation (DI), regulated deficit irrigation (RDI) and non-irrigated (NI)), the main compounds affected by water availability were proanthocyanidins and flavonols which were increased with irrigation at pea size, *veraison*, mid-ripening and full maturation phenological stages [128]. Concentrations of anthocyanin at full maturation were observed to be higher in the skin of berries belonging to DI and RDI vines than in NI ones. In general, although no differences in sugar accumulation were observed between the water treatments, a decrease in the quality parameters in grape skins in NI vines was observed, may resulting from high temperature and excessive cluster sunlight exposition.

## 4. Varietal Dependence on Grape Berry Phenolics

Traditionally, morphological and agronomical characteristics have been the main criteria for differentiating grapevine cultivars, but it is well known that many of those characters are strongly influenced by environmental conditions [130]. Grapevine varieties are not genetically homogeneous and intravarietal diversity varies across cultivars [131,132]. Even vines multiplied by vegetative propagation display a broad range of characteristics [133]. As referred to in the introduction, the grape phenolic profile depends greatly on the grape variety [7,36,134,135]. In a recent study, Liang *et al.* [20] showed that the polyphenol profile revealed significant differences among 344 European grape varieties. Polyphenol variations among several varieties are summarized in Table 3.

**Figure 5:**
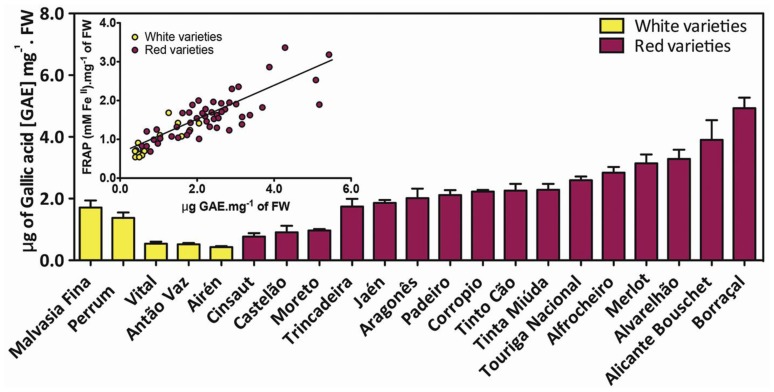
Total phenolic grape berry profile of 21 Portuguese *V. vinifera* varieties grown in Estremadura Region (Instituto Nacional de Investigação Agrária, INIA, Dois-Portos, Portugal), collected at full mature state. Error bars denote the SD from the mean, *n* = 3. Inset: correlation between total phenolic content and antioxidant activity (Teixeira, A., Eiras-Dias, J. and Gerós, H.).

Phenolics from grape and wine have generated remarkable interest with their antioxidant and free radical scavenging properties. Catechins, proanthocyanidins and anthocyanins are the most concentrated natural antioxidants present in red grape and wine [2,138] and it is believed that they play important beneficial roles in the mammalian systems [139]. The differences in phenolic composition observed across varieties might impact their respective health benefits. A study of 21 white and red winegrape varieties of Portugal showed remarkable differences in total phenolic concentrations in full mature berries, which were correlated to their total antioxidant activity (Figure 5). Of them, Borraçal grapes had the highest total phenolic content, even above the teinturier Alicante Bouschet.

Owing to its biological and agricultural importance, the genetics and biochemistry of the flavonoid biosynthetic pathway have been widely studied and the great intravarietal variability recommends the use of more precise methods to characterize and classify grape germplasm collections. Methods used to track back the variety and for producing a given wine rely on the composition in proteins, amino acids and aroma compounds, or on DNA analysis [130,140,141]. To a certain extent, flavonol profiles have demonstrated that some of them can be used as chemical markers for the authentication and varietal differentiation of grapes and wines [142]. Among those metabolic compounds, which have frequently been used as chemical markers in chemotaxonomy, in recent years the cultivar-characteristic profiles of monomeric anthocyanins have been widely used for the classification and differentiation of grape cultivars and monovarietal wines [38,143,144]. Despite the strong role of the genetic background in determining the composition of anthocyanins, the content of anthocyanins in grapes changes during their maturation and seasonal conditions, and the physical and chemical characteristics of the soil also influence the distribution of anthocyanins in grapes [130,136]. For example, Downey *et al.* [78] found that the anthocyanin fingerprint was altered by cluster exposition or shading to sunlight, by temperature regimes reached during the growing season, and by water deficit treatments [125]. Moreover, Guidoni *et al.* [145] stated that cluster thinning changed the proportion of anthocyanins, increasing cyanidin and peonidin 3-*O*-glucosides whereas malvidin 3-*O*-glucoside and acylated anthocyanins were not affected. The relative proportion of anthocyanins also varies during grape ripening; however, this composition is practically constant in the final stages of ripening [146]. Nevertheless, most references coincide with the fact that the non-genetic factors such as several environmental conditions or viticultural practices have a greater effect on the concentration of anthocyanins rather than on their relative composition [130,136]. Moreover, it is commonly accepted that anthocyanin concentration of grape berry also varies according to the genetic background, which is independent of seasonal conditions or production area [147].

## 5. Conclusions and Future Perspectives

Grapevine phenolics play distinctive roles during the development of the fruit until full maturation. Hydroxybenzoic acids may be involved in signaling, particularly in the induction of defense and stress responses, and stilbenes are effective antifungal agents. Flavonols are thought to act as UV and extreme temperature protectants, as well as free radical scavengers. The astringency role of proanthocyanidins (condensed tannins) is thought to act as a feeding deterrent to herbivorous and other insects. Anthocyanins play important roles in DNA protection and defense against photo-oxidative stress. In wine, hydroxycinnamates contribute to colour browning under oxidation in association with molecules. Also, proanthocyanidins contribute to mouthfell of red wine, as well as colour stability by forming complexes with anthocyanins that are responsible for the colour, and also contribute to the sensory attributes of wine. Important nutraceutical and pharmacologic properties have also been attributed to grape berry phenolics, including antimicrobial, anticarcinogenic and antioxidant. Several reports indicate that *trans*-resveratrol inhibits the proliferation of tumor cells and had a putative protection against diabetes. Their role against neurodegenerative diseases were recently postulated due to the resveratrol ability to activate the protein SIRT1 that was related to many diseases associated with aging [148]. Thus, the continued study of grape phenolics has an important basic and applied relevance.

The physiology of grapevine has already suffered from significant impacts of global climate change in recent decades. Harvest occurs sooner and sooner, although grape growers tend to wait longer for ripeness. Berry sugar content (and alcohol in the wine) tends to increase whereas phenolic and aromatic ripeness are not always achieved. Acidity tends to decrease with potential effects on wine aging capacity. Water supply is becoming shorter in many regions [149]. The site and season conditions are the most important factors that influence phenolic content of a grape cultivar. In particular, light and temperature affect to a great extent the phenolic content of the berry. These parameters are the most difficult to manage, although some viticulture practices, including strategic use of irrigation, utilization of cover crops, row orientation, trellising, and other canopy modifications may optimize plant interaction with light and temperature. Thus, the development of management strategies for optimizing grapevine phenolic composition in challenging environments is an important issue in modern viticulture. The improvement and implementation of standardized tools to quantitatively and qualitatively measure flavonoids in the grape berry is also an important research topic that could provide important developments in the future.

Although the inherent plasticity of grapevine response to environmental conditions may account for phenolic variation, several evidences introduced in this review show that phenolic profile is very dependent on the genotype. In this regard, the selection of new varieties with pleasant sensorial flavors but with improved climate tolerance may be an important investment for viticulturists and the wine industry. To address this challenge, scientists and breeders need to work together at an international level to generate knowledge about the valuable diversity, and patterns, processes and correlations with traits such as resistance and grape quality, which is the aim of the ongoing European Cost Action COSTFA1003 “East-West Collaboration for Grapevine Diversity Exploration and Mobilization of Adaptive Traits for Breeding” (2010–2013). For instance, despite the large number of studies on grape colour, there is still not a complete understanding of the genetics underlying this phenotype. In this regard, specific genes significantly associated with total skin and pulp anthocyanin were recently detected in red and rose cultivars from the Portuguese Ampelographic Collection, suggesting their involvement in anthocyanin content [150].

Important efforts have been undertaken by several research laboratories worldwide to understand and enhance the mechanisms of phenolic biosynthesis in grapevine, but this area of basic research is still widely open. Although the biosynthesis of many secondary compounds was already elucidated in some plants, the identification and characterization of specific transport steps have been published only recently, but a complete understanding of flavonoid transport and compartmentation in grape berry tissues in response to the environment is far from being elucidated. In addition, how the networks of phenolic biosynthesis are regulated and coordinated in different varieties, tissues and environments remains to be uncovered. In this regard, future investigation will involve the exploration of grapevine genetic diversity and the study of the role of specific genes or metabolic pathways in response to environmental conditions, taking advantage of the already available grapevine reference genome.

## Figures and Tables

**Table 1:** Phenolic compounds produced and accumulated in the grape berry [3,5–7,9,10,27–34].

Compound	Level of synthesis a			Location	Berry phenological scale b			
	Skin	Flesh	Seed		Blooming	Green stage	*Veraison*	Ripening
*Nonflavonoids*								
Hydroxycinnamic acids	++	+++	++	Hypodermal cells and placental cells of the pulp; primarily in the vacuoles of mesocarp cells.	+++	+++	+	+
Hydroxybenzoic acids	+	−	++					
Stilbenes	+++	+	++	Berry skin and seeds.	−	+	++	+++
*Flavonoids*								
Flavonols	++	−	−	Dermal cell vacuoles of the skin tissue and cell wall of skin and seeds.	++	+	+++	++
Flavan-3-ols	++	+	+++	Specific vacuoles of hypodermal skin cells and seed coat soft parenquima.	+	++	+++	++
Anthocyanins	+++	− *	−	Cell layers below the epidermis; storage confined to the vacuoles and cytoplasmic vesicles named anthocyanoplasts.	−	−	+	+++

a,b Very abundant compound (+++) to absent (−);

* Teinturiers contain anthocyanis also in mesocarp cells.

**Table 2:** Effect of water deficit on grapevine secondary metabolism.

Variety	Compound	Effect of water deficit	References
*Aragonez (Tempranillo)*	Anthocyanins	Decreased concentration.	[128]

*Barbera*	Resveratrol	No effect.	[117]

*Cabernet Sauvignon*	*Trans*-piceid stilbene precursors	5-fold increase in concentration. Increased transcript abundance of genes involved in the biosynthesis of stilbene precursors and phenylpropanoid metabolism in general.	[85,111,115,116,118,119,127,129]
	Flavonols	Increased concentration in the skin and in the wine. No changes in seeds.	
	Anthocyanins	Increased of concentration in the skin and in the wine.	
		Increased expression of many genes responsible for their biosynthesis.	

*Chardonnay*	Stilbene precursors	Increased concentration.	[119]
	Flavonols	Decreased transcript abundance of biosynthetic genes.	

*Merlot*	Anthocyanins	Increased concentration and biosynthesis;	[125,126]
	Proanthocyanidins	Increased concentration in berry skin.	

*Shiraz*	Anthocyanins	Increased concentration.	[124]

**Table 3:** Varietal differences in the grape berry composition.

Varietiy	Nonflavonoids			Flavonoids			References

	Hydroxycinnamic acids mg·g^−1^ FW	Hydroxybenzoic acids mg·g^−1^ FW	Stilbenes mg·g^−1^ FW	Flavonols mg·g^−1^ FW	Flavan- 3-ols mg·g^−1^ FW	Anthocyanins mg·g^−1^ FW	
*Araclinos*	0.742	0.034	0.001	0.042	0.386	0.655	[20]

*Aragonez*						0.658	[136]

*Cabernet*	0.103	0.011	0.003	0.039	1.830	1.830	[8,136,137]
*Sauvignon*			0.095			1.084	

*Chardonnay*	0.138	0.022			0.129		[20]

*Coudsi*	0.088	0.008	0.012	0.018	0.128		[20]

*Garnacha*						0.474	[137]

*Greco di Tufo*			0.0002				[7]

*Melon*	0.822			0.049			[20]

*Pinot Noir*	0.152	0.018	0.003	0.035	0.161	0.800	[7,20]

*Rofar Vidor*	0.402	0.081		0.053	0.440	0.655	[20]

*Royalty*			0.002	0.148	0.734	5.123	

*Sauvignon Blanc*	0.221	0.035	0.003	0.022	0.123		

*Touriga Nacional*	0.754	0.024	0.006	0.176	0.33	2.632	
